# Initial experience of robotic versus laparoscopic colectomy for transverse colon cancer: a matched case-control study

**DOI:** 10.1186/s12957-015-0708-1

**Published:** 2015-10-09

**Authors:** Nicola de’Angelis, Salah Alghamdi, Andrea Renda, Daniel Azoulay, Francesco Brunetti

**Affiliations:** Unit of Digestive, Hepato-Pancreato-Biliary Surgery, and Liver Transplantation, Henri Mondor Hospital, AP-HP, Université Paris Est, UPEC, 51, avenue du Maréchal de Lattre de Tassigny, 94010 Créteil, France; Department of Advanced Biomedical Sciences, University of Naples Federico II, 80125 Naples, Italy

**Keywords:** Transverse colon cancer, Transverse colectomy, Robotics, Robotic surgery, Laparoscopy, Case-control study

## Abstract

**Background:**

Robotic surgery for transverse colon cancer has rarely been described. This study reports our initial experience in robotic resection for transverse colon cancer, by comparing robotic transverse colectomy (RC) to laparoscopic transverse colectomy (LC) in terms of safety, feasibility, short-term outcomes, and the surgeon’s psychological stress and physical pain.

**Methods:**

The study population included the first 22 consecutive patients who underwent RC between March 2013 and December 2014 for histologically confirmed transverse colon adenocarcinoma. These patients were compared with 22 matched patients undergoing LC between December 2010 and February 2013. Patients were matched based on age, gender, body mass index (BMI), American Society of Anesthesiology (ASA) score, American Joint Committee on Cancer (AJCC) tumor stage, and tumor location (ratio 1:1). Mortality, morbidity, operative, and short-term oncologic outcomes were compared between groups. The operating surgeon’s stress and pain were assessed before and after surgery on a 0–100-mm visual analog scale.

**Results:**

The demographic and preoperative characteristics were comparable between RC and LC patients. No group difference was observed for intraoperative complications, blood loss, postoperative pain, time to flatus, time to regular diet, and hospital stay. RC was associated with longer operative time than LC (260 min vs. 225 min; *p* = 0.014), but the overall operative and robotic time in the RC group decreased over time reflecting the increasing experience in performing this procedure. No conversion to laparotomy was observed in the RC group, while two LC patients were converted due to uncontrolled bleeding and technically difficult middle colic pedicle dissection. Postoperative complications (Dindo-Clavien grade I or II) occurred in 11.3 % of patients with no group difference. Mortality was nil. All resections were R0, with >12 lymph nodes harvested in 90.9 % of RC and 95.5 % of LC patients. The surgeon’s stress was not different between RC and LC, whereas the surgeon’s hand and neck/shoulder pain were significantly lower after RC.

**Conclusions:**

RC for transverse colon cancer appears to be safe and feasible with short-term outcomes comparable to LC.

## Background

In the last decade, robotic surgery has gained increasing acceptance in colorectal surgery, showing good outcomes even after advanced procedures such as left and right colectomies, as well as after rectal resections [1–5]. A recent systematic review showed that robotic colorectal surgery is a safe and feasible option associated with similar outcomes in terms of oncologic results compared to laparoscopic or open surgery [1, 6].

The ability of robotics to overcome the natural limitations of traditional laparoscopy is promising and constitutes the main advantage of this emerging technology. However, robotic surgery has also several drawbacks such as the lack of haptic feedback, the bulky robotic cart, and the high costs, which might hamper the applicability of this technique to all colorectal procedures. In particular, most authors reported a longer operative time associated with robotic surgery, mainly attributable to the robotic setup and docking; moreover, technical difficulties have been correlated with specific colorectal procedures such as simultaneous splenic flexure mobilization and rectal resection, for which a multi-quadrant approach is required [1, 7].

Likely due to these reasons, robotic surgery has rarely been described for the resection of transverse colon cancers. Indeed, tumors located in the transverse colon have often been excluded even from randomized trials comparing laparoscopy and the open approach [8]. This is because of the complexity and risk of complications related to transverse colon cancer resection, which requires dissection in different quadrants of the abdominal cavity and the mobilization of both flexures. In addition, the proximity to adjacent structures such as the pancreas, duodenum, spleen, and the base of the mesenteric vessels represents a major risk of complication in case of dissection in the wrong plane. However, in recent years, few studies have demonstrated that laparoscopy is safe and feasible for transverse colon cancers, with the advantages associated with the minimally invasive approach, including less postoperative pain, faster recovery, and shorter hospital stay [8–12].

Even for experienced surgeons, performing complex procedures is known to induce high levels of psychological stress and fatigue. Mastering new technologies, such as robotic surgery, may decrease the surgeon’s stress in the operating room. Indeed, colorectal robotic surgery has been advocated as a technique with the advantage of a shorter learning curve compared to the traditional laparoscopy [13, 14], although little is known about surgery-related stress and surgeons’ preferences.

The aim of the present study was to report the initial experience of Henri Mondor Hospital in robotic colectomy for transverse colon cancer resection. Precisely, this study compares robotic and laparoscopic surgery in terms of safety, feasibility, short-term clinical outcomes, and the surgeon’s psychological stress and physical pain.

## Methods

### Patient selection

After obtaining approval from the institutional review board of the Henri Mondor Hospital, medical records were retrieved from a prospectively maintained database on patients who underwent colorectal surgery in the Unit of Digestive, Hepato-Pancreato-Biliary Surgery, and Liver Transplantation of Henri Mondor Hospital in Créteil, France. The study population included the first consecutive patients who underwent robotic transverse colon resections between March 2013 and December 2014 for histologically confirmed transverse colon adenocarcinoma. These patients were compared with a matched group of patients undergoing laparoscopic transverse colectomy between December 2010 and February 2013. Patients were matched based on age, gender, body mass index (BMI), American Society of Anesthesiology (ASA) score, American Joint Committee on Cancer (AJCC) tumor stage, and tumor location (ratio 1:1).

All procedures aimed for the curative resection of transverse colon cancer, defined as a tumor located between the hepatic and splenic flexures requiring ligation of the right or left branch of the middle colic artery (MCA) or both at their origins. Patients with ASA score >3, acute cancer obstruction requiring emergency surgery, or failure of self-expanding stent insertion; patients with evidence of cancer invading adjacent organs (AJCC seventh ed. tumor stage T4b); and patients requiring combined metastasectomy were excluded.

### Preoperative work-up

Before surgery, all patients underwent a comprehensive preoperative assessment. The tumor location was identified by colonoscopy and total body computed tomography (CT) with contrast enhancement. In case of suspected lymphatic packets, positron emission tomography (PET) with lymphatic biomarkers was performed for preoperative staging. For small lesions, Indian ink tattooing by colonoscopy was conducted 48 h before the surgical procedure. All patients received preoperative bowel preparation, parenteral antibiotics, and prophylaxis against deep venous thrombosis. Postoperative nasogastric tube was not routinely used. Conversely, a urinary catheter was always placed and then removed 24 h postoperatively.

### Operative technique

Laparoscopic-assisted transverse colon cancer resections were performed according to the standard techniques using a medial to lateral approach, as previously described [9, 12, 15].

**Fig. 1:**
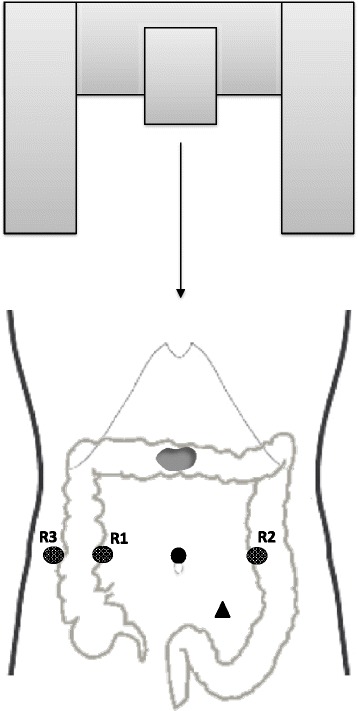
Schematic representation of robotic docking and port and cart placement for robotic transverse colectomy. The *filled black circle* represents the 12-mm camera port; the *three dotted circles* represent the 8-mm *R1*, *R2*, and *R3* robotic working arms; the *black triangle* represents the 12-mm assistant port; and the *gray irregularly shaped mass* represents the tumor

For robotic-assisted transverse colon cancer resections, the patient was placed in the lithotomy position with the knees at the level of the rest of the body and the arms alongside the body. Then, the table was placed in reverse Trendelenburg (15°). Four robotic ports were used, including one 12-mm camera port placed at the midline close to the umbilicus and three 8-mm robotic working ports. The first working port (R3) was placed as laterally as possible on the patient’s right side; the second (R1) and third (R2) ports were placed on the umbilicus line, on the right and left clavicular lines. One 12-mm port for the assistant surgeon was placed in the left lower quadrant, equidistant (approximately 10 cm) from the camera port and the robotic working port. The robotic cart was positioned over the patient’s head (Fig. 1). In all robotic procedures, a 30° optical camera was used. Based on the patient’s size and anatomy, working ports might have needed to be shifted; as a general rule, 8 to 10 cm should be maintained between all robotic ports.

Robotic transverse colectomy was approached by a medial to lateral dissection. Exploiting the peritoneal fixation of the right and left colon, the transverse mesocolon was lifted and spread by double fenestrated forceps through the R3 port and an atraumatic grasper through the assistant port. After obtaining an adequate exposure and identifying the middle colic vessels, the peritoneal layer of the mesentery was incised at the vessel pedicle origin. The dissection around the vessels was performed by monopolar curved scissors through the R1 port and fenestrated bipolar forceps through the R2 port. Once the middle colic artery and vein were isolated, they were ligated by a laparoscopic vascular stapler (Endo GIA®) through the assistant port. Then, the mesentery of the transverse colon was completely mobilized dividing the mesocolon from the root of the middle colic vessels to the border of the transverse colonic wall. The lateral attachments of the ascending and descending colon were dissected and both flexures were mobilized, leaving the transverse colon attached only to the gastro-colic ligament, which was divided at last.

Extracorporeal anastomosis and specimen removal were achieved by a mini-laparotomy using the Alexis Laparoscopy System® placed on the abdominal midline with a 4-cm incision superior to the umbilicus, for both robotic and laparoscopic procedures. All anastomoses were performed with a mechanical stapler (side to side).

The robot used in this study was the da Vinci Surgical System (Intuitive Surgical, Sunnyvale, CA, USA). All robotic and laparoscopic transverse colectomies were executed by two surgeons (FB, NdeA) experienced in minimally invasive colorectal surgery.

### Short-term outcomes

Demographic, perioperative, and short-term postoperative variables were prospectively recorded for analyses. Precisely, robotic and laparoscopic transverse colectomies were compared for operative time, intraoperative complication, conversion rate, blood loss, postoperative pain, time to flatus, time to regular diet, postoperative complication, length of hospital stay, and mortality at 30 and 60 days. Post-discharge follow-up lasted at least 60 days. The oncologic outcomes evaluated included the number of lymph nodes harvested and tumor-free margins.

### Surgeon’s psychological stress and physical pain

The assessment of the surgeon’s psychological stress and physical pain was introduced in 2012. Since then, the operating surgeon filled out four visual analog scales (VAS) for surgery-related stress, hand pain, neck/shoulder pain, and back pain just before (Pre-Op) and after (Post-Op) the procedure. A 0–100-mm VAS is used, with 0 indicating no stress/pain and 100 the maximal stress/pain ever experienced.

### Statistical analysis

The primary outcomes of the study were mortality and morbidity rates. Secondary outcomes included operative and postoperative variables and the surgeon’s psychological stress and physical pain. For comparisons between robotic (RC) and laparoscopic (LC) transverse colectomies, the chi-square test or Fisher’s exact test was used for categorical variables, and, according to the data distribution, the *T* test or Mann-Whitney *U* test was applied for continuous variables. Hence, continuous data were expressed as the means (standard deviation, SD) or medians and ranges (minimum to maximum). For VAS score analyses, repeated measures ANOVAs were performed with the surgical approach (RC or LC) as the between-subject variable and Pre-Op and Post-Op VAS scores as the within-subject variables. The Huynh-Feldt correction for sphericity was applied to all ANOVA calculations. A *p* value <0.05 was considered to be statistically significant. All statistical analyses were performed with SPSS (Statistical Package for Social Science, IBM SPSS Statistics, Version 22 for Macintosh).

## Results

### Surgical outcomes

Out of 382 colorectal procedures performed between December 2010 and December 2014, 22 consecutive patients who underwent RC were compared with 22 matched patients who underwent LC. The demographic, clinical, and preoperative characteristics of the study population are shown in Table 1. There were no significant differences between the RC and LC groups, which were matched by age, gender, BMI, ASA score, AJCC score, and tumor location. All patients had at least 60 days of postoperative follow-up.

**Table 1:** Demographic data and clinical characteristics of patients treated by robotic colectomy (RC) and laparoscopic colectomy (LC) for transverse colon cancer

Variables	RC group (*n* = 22)	LC group (*n* = 22)	*P* value
Gender (F/M) [n]	7/15	7/15	1
Age (years) [mean(SD)]	72.18(10.79)	71(10.14)	0.710
BMI (kg/m^2^) [mean(SD)]	24.12(2.64)	24.28(2.7)	0.841
Pre-operative hemoglobin (g/L) [mean(SD)]	11.67(1.6)	11.3(2.79)	0.916^a^
Pre-operative leukocytes (μL) [mean(SD)]	7522(1722)	6877(1599)	0.90
Serum albumin level (g/L) [mean(SD)]	37.52(3.78)	38.32(5.08)	0.577
Weight loss (>10 %) [n(%)]	3(13.6)	1(4.5)	0.607
Kidney failure [n(%)]	1(4.5)	1(4.5)	1
Diabetes [n(%)]	4(18.2)	1(4.5)	0.345
Cardiovascular diseases [n(%)]	8(36.4)	4(18.2)	0.310
Pulmonary disease [n(%)]	2(9.1)	2(9.1)	1
ASA score [n(%)]			1
I	9(40.9)	9(40.9)	
II	13(59.1)	13(59.1)	
III	0	0	
Previous abdominal surgery [n(%)]	3(13.6)	5(22.7)	0.698
TNM AJCC stage [n(%)]			1
I	1(4.5)	1(4.5)	
II	18(81.8)	18(81.8)	
III	3(13.7)	3(13.7)	

In TNM AJCC (7th ed.) categories II and III, the subcategories, including T4b tumors (*i.e.,* tumors directly invading or adhering to other organs or structures), were not included

*BMI* Body mass index; *ASA* American Society of Anesthesiology; *TNM* tumor, nodes and metastasis score; and *AJCC* American Joint Committee on Cancer. ^a^Mann-Whitney U Test

**Fig. 2:**
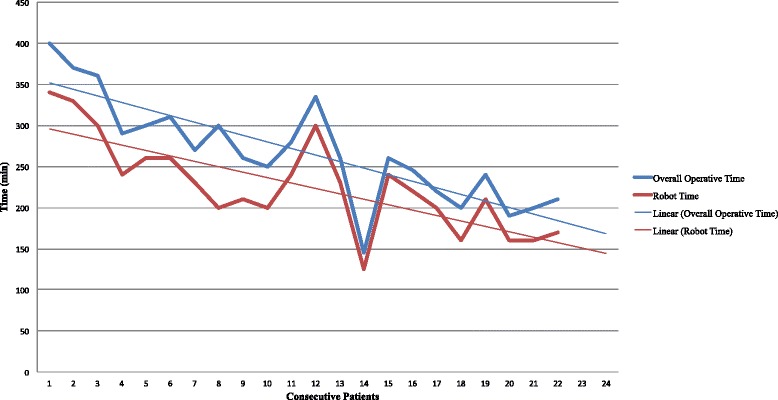
Overall operative time and robotic time trends (with linear forecast trend lines) of the first 22 consecutive patients operated on by robotic surgery for transverse colon cancer. The overall operative time includes the docking time, i.e., the installation of the robotic ports and cart, and the actual robotic time. The figure shows the tendency toward decrease of both robotic and overall operative times by the increasing surgeon experience in robotic transverse colectomy

Operatively, the two surgical approaches were comparable in terms of intraoperative complications, estimated blood loss, and the number of transfused patients (Table 2). Robotic procedures were associated with a longer median operative time (260 vs. 225 min; *p* = 0.014) compared with laparoscopy. For the RC procedure, the overall operative time and robotic time appeared to decrease over time (*p* = 0.03) (Fig. 2).

**Table 2:** Operative and postoperative outcomes of patients treated by robotic colectomy (RC) and laparoscopic colectomy (LC) for transverse colon cancer

Variables	RC group (*n* = 22)	LC group (*n* = 22)	*p* value
Overall operative time (min) [mean(SD and range)]	267.95(62.93) (145–400)	224.09(49.63) (160–360)	*0.014*
Robot time (min) [mean(SD and range)]	226.59(56.34) (125–340)		NA
Intraoperative complications [*n*(%)]	0	1(4.5)	1
Conversion to laparotomy [*n*(%)]	0	2(9.1)	0.488
Estimated blood loss (mL) [median (range)]	100(80–180)	110(60–200)	0.766^a^
Number of transfused patients [*n*(%)]	0	0	NA
Postoperative pain at day 1 (0–10 verbal scale) [mean(SD)]	4.72(1.16)	4.81(1.4)	0.772^a^
Postoperative pain at day 5 (0–10 verbal scale) [mean(SD)]	1.31(1.52)	1.63(1.53)	0.437^a^
Time to flatus [mean(SD and range)]	2.45(1.05)	2.41(0.79)	0.892^a^
	(1–4)	(1–4)	
Time to regular diet [mean(SD and range)]	4.04(1.17)	4.54(0.85)	0.072^a^
	(2–7)	(3–7)	
Postoperative complications [*n*(%)]	3(13.6)	2(9.1)	1
Type of complications			
Ileus	1	0	
Anastomotic leakage	1	1	
Intra-abdominal abscess	0	2	
Wound infection	1	0	
Pancreatic fistula	0	1	
Intestinal bleeding	0	0	
Dindo-Clavien classification [*n*(%)]			0.307
I	2(9.1)	0	
II	1(4.5)	2(9.1)	
Length of hospital stay (days)[mean(SD and range)]	7.09(1.68) (5–13)	8.09(2.15)(6–15)	0.145^a^
Mortality within 30 days [*n*(%)]	0	0	NA
Mortality within 60 days [*n*(%)]	0	0	NA
Re-admission within 60 days [*n*(%)]	1(4.5)	2(9.1)	1

The overall operative time includes the port positioning time, laparoscopic surgical time, robot docking time, and robot surgical time until all surgical incisions are sutured. The robot time quantifies the robot surgical time only (without the port positioning and robot docking times)

^a^Mann-Whitney *U* test

P values in italics denote significant differences between groups

NA stands for not applicable

In the LC group, two patients were converted to laparotomy due to uncontrolled bleeding during middle colic pedicle dissection in one case and technically difficult middle colic pedicle dissection in the other. No conversion to laparotomy was observed in the RC group.

Postoperatively, no group difference was observed in terms of postoperative pain (at either day 1 or day 5), time to flatus, time to regular diet, or length of hospital stay (Table 2). Overall, five patients presented with postoperative complications, with no difference between groups (three in the RC group, two in the LC group). All complications were class I or II based on the Dindo-Clavien classification and were medically managed, except for one patient with pancreatic fistula who was treated by CT-guided percutaneous drainage. Mortality at 30 and 60 days follow-up was nil. Only three patients (one in the RC group, two in the LC group) were re-admitted within 60 days following surgery; reasons for re-admission included wound and urinary infections.

Histologically, all resections were defined as R0 (Table 3). The mean number of lymph nodes harvested did not differ between groups (17.45 ± 5.44 in the RC group vs. 19.13 ± 3.39 in the LC group; *p* = 0.081), with more than 12 lymph nodes harvested in the majority of patients (90.9 % in the RC group, 95.5 % in the LC group). The tumor size and grade of differentiation did not differ between groups.

**Table 3:** Histologic findings in patients treated by robotic colectomy (RC) or laparoscopic colectomy (LC) for transverse colon cancer

Variables	RC group (*n* = 22)	LC group (*n* = 22)	*p* value
R0 resection [*n*(%)]	22(100)	22(100)	NA
Number of lymph nodes harvested [*n*(%)]			1
<12 lymph nodes	2(9.1)	1(4.5)	
≥12 lymph nodes	20(90.9)	21(95.5)	
Tumor size max diameter (cm)[mean(SD and range)]	4.68(1.93)(0.5–8.5)	4.5(1.81)(1–8)	0.749
Adenocarcinoma [*n*(%)]			0.582
Well differentiated	10(45.4)	13(59.1)	
Moderately differentiated	8(36.4)	5(22.7)	
Mucinous	4(18.2)	4(18.2)	

NA stands for not applicable

### Surgeon’s psychological stress and physical pain

The surgeon’s subjective assessment of psychological stress and physical pain before and after RC and LC is displayed in Table 4. VAS questionnaires were available for 24 surgeries only (12 RC and 12 LC). The mean surgery-related stress did not differ between the RC and LC procedures, although a significant increase in the stress level was observed after surgeries using either approach. A significant group difference was observed for hand and neck/shoulder pain, which were judged to be at a lower intensity after robotic procedures compared with laparoscopic ones.

**Table 4:** Measures of psychological stress and physical pain related to robotic (RC) and laparoscopic (LC) surgeries, assessed by the operating surgeon on a 0–100-mm visual analog scale (VAS) before (Pre-Op) and after (Post-Op) the procedure

VAS score	RC (*n* = 12)		LC (*n* = 12)		*p* value	
	Pre-Op	Post-OP	Pre-Op	Post-OP	Within subjects	Between subjects
Psychological stress [mean(SD)]	49.5(9.3)	53.2(14.1)	55.6(6.4)	63.9(5.7)	*0.045*	0.550
Hand pain [mean(SD)]	14.7(5.5)	39.2(8.2)	18.3(5.9)	52.7(8.4)	*0.0001*	*0.0001*
Neck and shoulder pain [mean(SD)]	23.2(11.1)	28.8(12.3)	23.5(9.7)	42.9(7.4)	*0.0001*	*0.0001*
Back pain [mean(SD)]	21.2(8.9)	44.8(10.3)	26.4(8.3)	43.5(9.1)	*0.0001*	0.488

P values in italics denote significant differences between groups

## Discussion

The present study shows that robotic colectomy for transverse colon cancer can achieve complete resection and good short-term oncological results that are comparable to laparoscopy. Both minimally invasive surgical techniques are safe and associated with similar favorable postoperative outcomes in terms of morbidity and patient’s pain and recovery. Within the limitations of the study, robotic surgery appears to bring advantages to the operating surgeon, who experienced less physical fatigue and pain when performing robotic rather than laparoscopic approach.

Robotic surgery has not been widely applied for interventions requiring a wide range of motion of the robotic arms or a multiple-quadrant surgical field. The present study demonstrated that a single-docking and single-stage robotic approach is safe and feasible for transverse colon cancer resection. Based on the tumor location, which needs to be precisely assessed preoperatively by CT scan and ink tattooing, robotic docking is optimized for the type of intervention without the need for time-consuming intraoperative re-docking or re-staging. However, as described in the literature [1, 4, 6], also in the present study, the operating time of robotic procedures was significantly longer than that of laparoscopic ones. This increased operating time is actually due to the docking time, i.e., the installation of the robotic ports and cart, which, however, shows a linear decrease over time reflecting the increasing robotic surgical experience. Thus, it may be expected that at the end of the learning curve, the differences in operating time between laparoscopy and robotic surgery might become insignificant and negligible. Moreover, it is worth noting that the advantages of the minimally invasive approach are maintained while performing robotic surgery despite the increased operating time.

The achievement of adequate lymphadenectomy and R0 resection by laparoscopic transverse colectomy has been questioned because the dissection of regional lymph nodes around the middle colic artery can be difficult [16, 17]. However, the present study supports that this is technically feasible with both laparoscopic and robotic approaches, by which a similar and sufficient number of lymph nodes (>12) was harvested [18]. Surely, the dissection of the middle colic artery represents the most critical step of transverse colectomy. In the present study, conversion to laparotomy was required for two patients in the LC group due to difficulties in middle colic pedicle dissection. No conversion was required for RC. Although not statistically significant between the two groups, the 0 % conversion rate for RC may be related to the three-dimensional, high-definition, steady robotic camera that counterbalances the lack of haptic feedback. In all robotic procedures, the vascular pedicle resection was achieved by vascular Endo GIA® through the assistant port. The EndoWrist® GIA Stapler is not yet available in our unit, but its use could certainly contribute to an easier and safer management of vascular section. Moreover, in the case of the integrated EndoWrist® stapler, a 5-mm assistant port could be used only for traction, with the resulting benefit of less postoperative pain.

The present results also show that the operating surgeon experienced significantly lower surgery-related hand and neck/shoulder pain when operating with the robotic technique compared with the laparoscopic one. This is surely due to the high ergonomic comfort of the robotic console and the proper hand-eye coordination. Indeed, the surgeon sits at a remote, ergonomically designed robotic workstation without needing to move, turn, or twist in awkward positions to use the robotic arms. Moreover, the surgeon’s psychological stress related to the application of a new technology in a technically demanding operation (i.e., transverse colectomy) appeared not to be different from that reported for laparoscopy. To the best of our knowledge, this is one of the first studies to assess the surgeon’s psychological stress and physical pain between two surgical approaches, laparoscopy and robotic surgery. Despite the paucity of data, it seems obvious that the surgeons’ overall well-being would benefit from optimization of the surgical environment, which may have an impact on the perceived level of stress, as well as on the surgical performance, ultimately resulting in improved patient outcomes [19]. However, in the present study, the surgeon’s distress was not assessed by means of truly objective measures (e.g., heart rate, serum levels of stress-related factors [20]), and thus, further studies are awaited to clarify this facet. Moreover, these results must be interpreted with caution due to the limited sample size and the non-randomized study design (no power analysis), and they cannot be generalized since they describe the initial experience in robotic transverse colectomy of a surgical team accustomed to robotic colorectal surgery [21, 22].

As known, robotics has been introduced in colorectal surgery to possibly overcome some of the laparoscopic drawbacks and improve steadiness, precision, dexterity, and ergonomy [3, 5, 23]. Indeed, although laparoscopy has been widely proven to be safe, feasible, and as effective as open surgery for the resection of colorectal cancers [24–26], it remains technically challenging [25, 27]. Conversely, robotics seems to be associated with a shorter learning curve, which may ease some surgical procedures and favor its widespread applications. However, this is true only in theory because the high costs associated with acquisition and maintenance have drastically limited the implementation of robotic platforms [28]. In a cost-effectiveness evaluation, laparoscopy still scores over robotic surgery, although this has never been analyzed taking into account the level of surgical experience, the different colorectal procedures, the short- and long-term oncologic outcomes, as well as the surgeons’ preferences. It may be too early to draw definitive conclusions on robotic colorectal surgery solely based on the early experience of few specialized centers [29]. Definitely, further prospective studies are required to evaluate the outcomes of robotic colorectal surgery. Moreover, it would be interesting to use simulation models to determine the cost-effectiveness of this new technology over a 10- or 20-year period prior to the acceptance of robotics as the preferred approach.

## Conclusions

Our early experience suggests that robotic colectomy for transverse colon cancer is safe, feasible, and associated with short-term outcomes that are comparable to laparoscopy. At the level of the operating surgeon, the robotic approach appears to be associated with similar psychological stress but greater comfort.

## Abbreviations

AJCC: American Joint Committee on Cancer
ASA: American Society of Anesthesiology
BMI: body mass index
LC: laparoscopic colectomy
RC: robotic colectomy
TNM: tumor, node, and metastasis score
